# Dysfunction of *Drosophila* mitochondrial carrier homolog (Mtch) alters apoptosis and disturbs development

**DOI:** 10.1002/2211-5463.13742

**Published:** 2023-12-19

**Authors:** Cristina González, Lidia Martínez‐Sánchez, Paula Clemente, Janne Markus Toivonen, Juan José Arredondo, Miguel Ángel Fernández‐Moreno, José Alberto Carrodeguas

**Affiliations:** ^1^ Departamento de Bioquímica & Instituto de Investigaciones Biomédicas “Alberto Sols” The Autonomous University of Madrid‐Consejo Superior de Investigaciones Científicas Spain; ^2^ LAGENBIO, Departamento de Anatomía, Embriología y Genética Animal, Facultad de Veterinaria, Instituto Agroalimentario de Aragón (IA2) Universidad de Zaragoza Spain; ^3^ IIS Aragón Zaragoza Spain; ^4^ Centro de Investigación Biomédica en Red de Enfermedades Neurodegenerativas (CIBERNED) Madrid Spain; ^5^ Centro de Investigación Biomédica en Red en Enfermedades Raras (CIBERER) Facultad de Medicina, UAM Madrid Spain; ^6^ Departamento de Bioquímica & Instituto de Investigaciones Biomédicas Sols‐Morreale The Autonomous University of Madrid‐Consejo Superior de Investigaciones Científicas Madrid Spain; ^7^ Institute for Biocomputation and Physics of Complex Systems (BIFI) University of Zaragoza Spain; ^8^ Department of Biochemistry and Molecular and Cellular Biology, School of Sciences University of Zaragoza Spain

**Keywords:** apoptosis, development, *Drosophila*, mitochondria, mitochondrial carrier homolog (MTCH)

## Abstract

Mitochondrial carrier homologs 1 (MTCH1) and 2 (MTCH2) are orphan members of the mitochondrial transporter family SLC25. Human MTCH1 is also known as presenilin 1‐associated protein, PSAP. MTCH2 is a receptor for tBid and is related to lipid metabolism. Both proteins have been recently described as protein insertases of the outer mitochondrial membrane. We have depleted Mtch in *Drosophila* and show here that mutant flies are unable to complete development, showing an excess of apoptosis during pupation; this observation was confirmed by RNAi in Schneider cells. These findings are contrary to what has been described in humans. We discuss the implications in view of recent reports concerning the function of these proteins.

## Graphical abstract

Mitochondrial carrier homologs 1 (MTCH1) and 2 (MTCH2) are outer mitochondrial membrane translocases related to inner membrane carriers. In humans, MTCH1 interacts with presenilin 1 and induces apoptosis upon overexpression. We show here that the *Drosophila* ortholog is required for development, inducing apoptosis upon its downregulation both in the whole organism and in cultured cells.

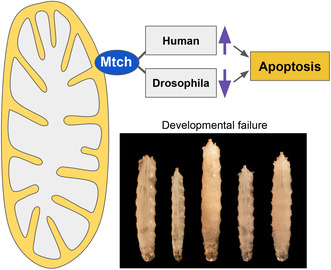

**Abbreviations**
APP: amyloid precursor protein
BAK: BCL‐2 homologous antagonist/killer
BAX: BCL‐2‐associated (protein) X
BID: BH3 interacting‐domain death agonist
tBID: truncated BID
Debcl: death executioner BCL‐2
DR6: death receptor 6
ER: endoplasmic reticulum
dsRNA: double‐stranded RNA
HGF/SF: hepatocyte growth factor/scatter factor
Met: protein kinase Met
MIMP: Met‐induced mitochondrial protein
MTCH: mitochondrial carrier homolog
PSAP: presenilin 1‐associated protein
PSAPL: large isoform of PSAP
PSAPS: short isoform of PSAP
SMAC: second mitochondria‐derived activator of caspase

Apoptosis is a type of programmed cell death required for development and tissue homeostasis in multicellular organisms and therefore involved in several pathologies. In mammals, two major apoptotic pathways have been described, one involves mitochondria as a reservoir of proteins which trigger a cascade of apoptotic events, the so‐called intrinsic pathway [1], while the other depends on cell surface receptors which are part of the extrinsic or death receptor pathway [2]. In some cells, both pathways are connected by BID, a protein that, upon cleavage by extrinsic pathway‐activated cysteine‐aspartate proteases (caspases), translocates to mitochondria as truncated BID (tBID) to activate the intrinsic pathway [3, 4]. At the outer mitochondrial membrane tBID interacts with MTCH2 [5], a protein belonging to the mitochondrial inner membrane carrier family SLC25 [6]. MTCH2 was initially reported as MIMP (Met‐induced mitochondrial protein; [7]), a protein whose expression increases when hepatocyte growth factor (HGF) binds to its specific receptor at the cell surface. MTCH2 knockout in mice has shown that it is required to recruit tBID to mitochondria [5], being, therefore, involved in apoptosis triggering. MTCH2 also modulates the body mass index [8], likely through lipid metabolism [9], being reported as a regulator of lipid homeostasis that influences the activity of estrogen receptor 1 [10]. Landgraf *et al*. [11] had previously shown that MTCH2 is involved in liver and intestine development in zebrafish, likely through alteration of Met‐HGF/SF signal transduction. In pigs, modification of *MTCH2* mRNA by N^6^‐methyladenosine (m^6^A) promotes adipogenesis in intramuscular preadipocytes [12]. Labbé *et al*. [13] reported that MTCH2 is a regulator of mitochondrial fusion in response to lysophosphatidic acid, inducing hyperfusion in response to starvation, suggesting that it may be antagonizing the function of another outer membrane protein of the SLC25 family, SLC25A46 [14], which participates in lipid transfer between the ER and mitochondria, being involved in Leigh syndrome [15, 16]. Despite its sequence homology with inner membrane transporters no function in metabolite transport has been so far assigned to MTCH2. This is due to the lack of the charged amino acids that form salt‐bridges, essential for metabolite transport in these carrier‐like outer membrane proteins [17, 18].

MTCH1, a MTCH2 homolog, was initially reported as a presenilin‐1 interacting protein (PSAP) [19]. Presenilin‐1 is a component of the gamma secretase complex and is involved in the proteolysis of several integral membrane proteins such as amyloid precursor protein (APP), being therefore a focus of interest for Alzheimer's disease research [20]. MTCH1 has two major isoforms, PSAPL and PSAPS, generated by alternative splicing. Both contain two apoptotic domains that can individually induce apoptosis when overexpressed at the outer mitochondrial membrane [21, 22]. In 2013, Vural *et al*. [23] reported the presence of antibodies against MTCH1 in neuro‐Behçet's disease, an inflammatory disorder, and in 2021, it was described that MTCH1 peptides are present in plasma from patients with sepsis [24]. MTCH1 is able to induce apoptosis in a manner not regulated by BCL‐2 family proteins [25], although it requires SMAC [26]. In addition, Zeng *et al*. [27] showed that MTCH1 mediates presenilin 1‐induced apoptosis in a γ‐secretase‐independent manner forming a complex with BAX. These authors suggest that MTCH1 functions as a receptor or anchor for BAX under certain apoptotic conditions when MTCH1 is present at endogenous levels, although it is able to induce apoptosis in a BCL‐2 protein‐independent manner when it is overexpressed [25, 26]. In 2020, the same research group reported the participation of MTCH1 in apoptotic death induced by activation of the DR6 death receptor [28]. Rottiers *et al*. [10] reported the effects of altering the levels of *Caenorhabditis elegans* MTCH (referred to as MTCH‐1 in their report) in lipid accumulation, causing developmental and fertility problems in the worm. Chen *et al*. [29] recently reported that MTCH1 upregulation is associated with cell proliferation, invasion, and migration of liver hepatocellular carcinoma, and suggested a possible role for MTCH1 in RNA splicing, although they did not comment on how a mitochondrial outer membrane protein could be involved in a process that takes place inside the nucleus. Very recently, as this manuscript was being prepared, Guna *et al*. [30] reported that MTCH2 is an outer membrane protein insertase.

To further elucidate the function of these proteins in whole organisms, we have used fruit fly *Drosophila melanogaster* as a model system. *Drosophila* has two putative *Mtch1* orthologs named CG6851/*Mtch* and CG10920. However, it was not clear how these relate to human MTCH1 and MTCH2 or, indeed, if both are likely to encode functional *Mtch* orthologs. In this work, we first carried out phylogenetic analysis and looked at the tissue‐level expression profile of both putative *Drosophil*a Mtch orthologs. Then, we studied the role of Mtch in *Drosophila*, analyzing its knock down in cultured cells as well as characterizing in the whole animal the development and survival capabilities of two different P‐element insertional mutants. Our results indicate that (a) CG6851 is likely the major *Mtch* ortholog in flies with CG10920 devoted to a testis‐specific function, (b) Mtch is essential for proper fly development, (c) apoptosis is increased in homozygous mutant imaginal discs, and (d) reducing Mtch levels in cultured cells increases apoptosis. These results show unprecedented evidence on the induction of apoptotic signals upon depletion of *Drosophila* Mtch, contrary to previous observations in mammals, associated with improper fly development.

## Materials and methods

### *Drosophila* mutant strains and genetics

*Drosophila melanogaster* mitochondrial carrier homolog 1, *Mtch1* (CG6851) is located on chromosome arm 3L. Mutant lines *Mtch* ^G20854^ and *Mtch* ^G8642^ were obtained from the Bloomington *Drosophila* Stock Center (https://bdsc.indiana.edu/, Table 1). P‐element mobilization was performed following standard procedures [31]. Line Δ 2‐3 (y[1] w[*]; ry[506] Sb[1] P{ry[+t7.2] = Delta2‐3}99B/TM6)(BI3664) [32] was a kind gift from M. Calleja (Centro de Biología Molecular Severo Ochoa, Universidad Autónoma, Madrid, Spain). Revertants were identified based on eye color, once the chromosome had segregated and there was absence of phenotype. PCR around the insertion zone of each of the mobilized P elements was carried out to confirm the absence of the element as well as other defects derived from the mobilization. At least six revertants were tested for each of the lines.

**Table 1:** Specifications of *Drosophila* mutants used in this work.

Stock ID^a^	Stock no.^b^	Genotype	Genomic insertion	Insertion with respect to *Mtch* gene
G8642	27981	y^1^ w*; P{EP} Mtch^G6842^/TM3, Sb^1^, Ser^1^	3L, base 185035	Coding sequence: 400 bases downstream of transcription start site
G20854	28432	y^1^ w*; P{EP} Mtch^G20854^/TM3, Sb^1^, Ser^1^	3L, base 184663	5′UTR: 28 bases downstream of transcription start site

^a^ Refers to the identification number of the specific P element insertion.

^b^ Refers to the number identifying the stock in the Bloomington *Drosophila* Stock Center.

### Phylogenetic analysis

Sequences of *Mtch* orthologs from different species, including 12 sequenced drosophilids (Table S1), were aligned using clustal omega and phylogenetic tree files were constructed using clustalw2 phylogeny (EMBL‐EBI). The trees were constructed from Newick files using itol [33].

### Imaginal disc analysis

Wing imaginal disc staining and analysis was performed as described in [34], staining actin in red and activated caspase 3 in green. Nuclei were stained with ToPro3 (Thermo Fisher Scientific, Waltham, MA, USA) following instructions provided by the manufacturer, rabbit anti‐caspase 3 (Cell Signaling Technology, Danvers, MA, USA) was used at 1 : 50 dilution and detected with Alexa Fluor 488‐conjugated donkey anti‐rabbit antibody (Thermo Fisher Scientific, Waltham, MA, USA) at 1 : 2000 dilution. Actin was detected with Alexa Fluor 647‐conjugated phalloidin (Thermo Fisher Scientific, Waltham, MA, USA) at 1 : 50 dilution.

### Schneider S2 cell culture

Schneider S2 cells were maintained in *Schneider Drosophila Medium* (Thermo Fisher Scientific #21720‐024, Waltham, MA, USA) supplemented with 10% Fetal Bovine Serum (Thermo Fisher Scientific Corp., Waltham, MA, USA), penicillin G (100 IU·mL^−1^), and streptomycin (100 mg·mL^−1^) at 25 °C. Cells were diluted 1 : 4 twice a week.

### Mtch mRNA interference assay

Knock‐down of *D. melanogaster Mtch* gene (GenBank ID: 23787) was carried out in S2 cells according to Fernandez‐Moreno et al. [35] with some modifications. dsRNA internal to *Mtch* mRNA was *in vitro* transcribed using the *MEGAscript T7 transcription kit* (Thermo Fisher Scientific, Waltham, MA, USA) using as template a 272 pb in size PCR‐derived fragment (222 bp corresponding to nucleotides 254–476 of *Mtch* mRNA, plus T7 promoter sequences at each end) carrying the T7 promoter sequence at both ends (underlined): DMPSAPT7PROM1F‐5′‐GAATTAATACGACTCACTATAGGGAAGCACGCCCGCGCAGAGGAT‐3′ as forward primer and DMPSAPT7PROM1R‐5′‐GAATTAATACGACTCACTATAGGGAAGCCGTCGATCCGCCGAATG‐3′ as reverse primer. As a negative control, a 219 pb in size PCR‐derived fragment flanked by the T7 promoter sequence (underlined) was also used as template to *in vitro* transcribe a dsRNA internal to bacterial *lacZ* gene. In this case, primers used were: T7 LacZ FW‐5′‐GAATTAATACGACTCACTATAGGGAGATCATGGTCATAGCTGTTTCCT‐3′ as forward primer andT7 LacZ RV‐5′‐GAATTAATACGACTCACTATAGGGAGAAACCGCCTCTCCCCG‐3′ as reverse primer.

For RNA interference, 30 μg of dsRNA were added directly to 4 × 10^6^ S2 cells exponentially growing into 2 mL of *Schneider Drosophila Medium* supplemented with 10% fetal bovine serum without antibiotics. Plate was swirled by hand and cells were incubated at 25 °C for 1 h. Then, 3 mL of fresh complete medium were added. Twenty‐four hours later, cells were harvested by centrifugation (240 ***g***, 5 min), washed with PBS and resuspended in 5 mL of fresh complete media followed by an additional incubation at 25 °C for 48 h. Then, cells were spread out on three plates for the apoptosis assay, staurosporine treatment before apoptosis assay and RNA extraction and quantification.

The level of *Mtch* mRNA was analyzed by real‐time quantitative PCR using three different pairs of primers:

Dm‐qmtch1‐FW‐5′‐GGTTAATGTTTGGATCCGCTTT‐3′ with Dm‐qmtch1‐RV‐5′‐AACCCACCAATTTAGGAGCTAGAC‐3′, Dm‐qmtch2‐FW‐5′‐CAGCTCCCTTTTCTGGCGGTCA‐3′ with Dm‐qmtch2‐RV‐5′‐CATACCGGAGCCCGTCCGTG‐3′ and Dm‐qmtch3‐FW‐5′‐GCTCCCTTTTCTGGCGGTCAC‐3′ with Dm‐qmtch3‐RV‐5′‐GTCCGTGGTCGTCCAGCCTC‐3′. For normalization of *Mtch* mRNA levels, quantification of the mRNA from three different genes was made using the following pairs of primers:

For *Alpha‐tub84B* mRNA (FBtr0081639) primers aDmTub2‐FW‐5′‐GGCATGGACTCCGGTGACGG‐3′ and aDmTub2‐RV‐5′‐GCCCACCAATGACGCTCCCA‐3′ were used. For *Actin42A* mRNA (FBtr0086029) primers Actin42A2‐FW‐5′‐GCCGCTTCAAGCTCGTCCCT‐3′ and Actin42A2‐RV‐5′‐AGCGATTCGGGGCAACGGAA‐3′ were used. Finally, for the mRNA encoding the *Polr2B* (RNA polymerase II 140 kDa subunit; FBgn0262955) primers DmRpII140‐FW2‐5′‐GGGCGTGTGCGCGTCCATTA‐3′ and DmRpII140‐RV2‐5′‐AAACGCCCATAGCTTGCTTACCCAT‐3′ were used. *Mtch* mRNA interference were considered as the media of the measures of the three detectors (Dm‐qmtch1, Dm‐qmtch2 and Dm‐qmtch3) normalized with *Alpha‐tub84B, Actin42A* and *Polr2B*.

RNA was extracted with Trizol reagent (Merck, Darmstadt, Germany), and cDNA synthesized using the QuantiTect Reverse Transcription Kit (Qiagen, Venlo, The Netherlands), followed by SYBR green RTqPCR using an Applied Biosystems Step‐One Plus real‐time thermocycler (Thermo Fisher Scientific, Waltham, MA, USA), consisting of an initial 10 min denaturation step at 95 °C followed by 40 cycles of denaturation (15 s at 95 °C) and annealing/extension (1 min at 60 °C). The *Mtch* mRNA relative levels were determined by qPCR, normalizing to Alpha‐tub84B (FBtr0081639), Actin42A (FBtr0086029) and Polr2B (RNA polymerase II 140 kDa subunit; FBgn0262955) mRNA levels and showing their media and standard deviation values.

### Apoptosis assays by flow cytometry

For apoptosis assays, 7.5 × 10^5^ S2 cells were treated with *Mitostep + Annexin V‐FITC Apoptosis Detection Kit* (Immunostep ref.: KMAF‐100T) following manufacturer recommendations and processed by flow cytometry. In some cases, apoptosis was induced by 5 μm Staurosporine for 20 h before the analysis. The fluorescence intensity of at least 10 000 events was determined in a FACScan cytometer (Cytomics FC 500MPL Beckman Coulter) using mpx (Beckman Coulter, Brea, CA, USA) acquisition software. Data were obtained as the media of six different experiments and their corresponding standard deviation.

### Quantification and statistical analysis

Statistical details of the experiments including statistical tests used, exact value of *n*, dispersion and precision measures (mean ± SD), and statistical significance are reported in the Figures and Figure Legends. The differences between two groups were analyzed using a two‐tailed Student's *t*‐test. All statistical analyses were performed using graphpad prism 8 software, La Jolla, CA, USA. A *P* value lower than 0.05 was considered significant.

## Results

### *Drosophila melanogaster* Mtch orthologs

In order to identify *Drosophila* orthologs of the human proteins in sequence databases, we carried out a protein blast (National Center for Biotechnology Information) search using human MTCH1 protein sequence (large isoform, PSAP‐LS, NP_055156.1) against *Drosophila melanogaster* protein databases, which identified NP_523869 as the *Drosophila Mtch1* ortholog, annotated in GenBank as mitochondrial carrier homolog 1, variant A (*Mtch*, nucleotide sequence accession number NM_079145, corresponding to locus Dme1 CG6851). When this protein is compared with human MTCH1, it shows a total score of 196, an E value of 1e‐59, 37% identities and 57% positives. The next sequence identified, in order of homology, is annotated as CG10920 and displays a total score of 117, an E value of 3e‐29, 28% identities and 44% positives.

**Fig. 1:**
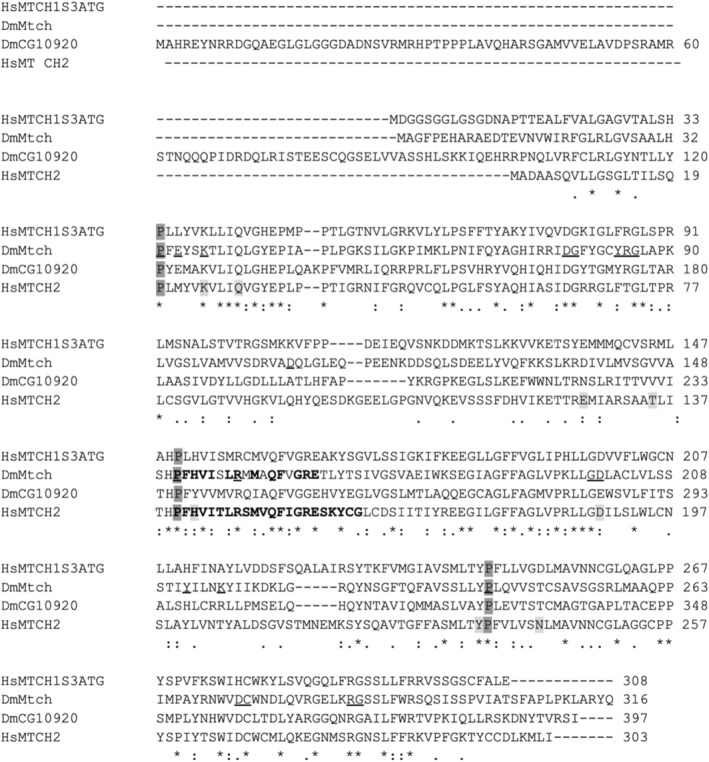
Alignment of human and *Drosophila* proteins. Human Mtch1 (short spliced isoform starting at the third ATG, HsMTCH1S3ATG), human Mtch2 (HsMTCH2), *Drosophila* Mtch (DmMtch) and *Drosophila* CG10920 (DmCG10920) were aligned using clustalw2 with default settings. An asterisk indicates fully conserved residues; colon indicates strongly similar properties; period indicates weakly similar properties. Amino acids in bold indicate a peptide in human Mtch2 (140–161) that binds tightest to tBid [53]. Equivalent amino acids conserved in DmMtch are also marked in bold letters. Underlined amino acids in the DmMtch sequence refer to those that have been reported by Robinson *et al*. [17] to be conserved in mitochondrial carrier proteins, specifically referring to the *Bos taurus* ATP/ADP carrier shown in their Fig. 1. The proline (dark gray) that is part of the Px[D/E]xx[K/R] motif present in the SLC25 repeats, as well as polar and charged residues (light gray) highlighted by Guna *et al*. [30] are also marked in the human MTCH2 sequence.

Assignment of CG6851 and CG10920 to Mtch1 or Mtch2 is not trivial when just based on sequence homology, as concluded after several sequence alignments, summarized in Table 2. We must recall that human MTCH1 has two isoforms generated by alternative splicing that differ from each other in a stretch of 17 amino acids in an internal region of the protein, in such a way that we named PSAPS and PSAPL the short and large isoforms respectively [21]. Both isoforms have three putative translation start codons. Xu *et al*. [22] reported that PSAP is mainly translated from the second ATG, nevertheless alignment with *Drosophila* Mtch (CG6851, referred to as Mtch from now on) reveals that this protein is shorter than human MTCH1 and its amino terminal end aligns better with amino acids starting at the third ATG in human MTCH1, i.e., Mtch lacks amino terminal amino acids with respect to human MTCH1. Therefore, we also include MTCH1 protein sequences starting at the third ATG in Table 2 (Human PSAPS 3ATG) and use this protein in the alignments shown in Fig. 1 (HsMTCH1S3ATG). Since MTCH1 has internal localization signals these alternative initiation sites do not affect mitochondrial localization [21, 22]. It can be concluded that both human MTCH1 and human MTCH2 have higher similarity and identity with Mtch than with CG10920. Mtch was considered as the *Drosophila* Mtch2 homolog by Grinberg *et al*. [36]. *Drosophila* Mtch proteins do not have a high similarity between them, indicating that conserved amino acids among each of them and the mammalian proteins are different from the ones conserved between both *Drosophila* proteins (Fig. 1). Since Robinson *et al*. [17] reported key amino acids in the mitochondrial carrier family and pinpointed some specific features of human MTCH2 sequences, we have marked those amino acids in Fig. 1 (Mtch sequence, underlined amino acids). It can be observed that most of those amino acids are conserved among all four proteins. In order to determine which of the two *Drosophila* proteins could be more important for fly physiology, we carried out a deeper analysis of these two proteins in drosophilids (see Table 3) and other organisms.

**Table 2:** Similarities and identities between human and *Drosophila* Mtch proteins. Similarity refers to the number of identical amino acids plus the number of similar amino acids in the same positions. Identity refers to the number of identical amino acids in the same positions. Both are expressed as a percentage with respect to the total number of amino acids in the protein. PSAPL and PSAPS refer to the larger isoform and the shorter one of human Mtch1, both starting at the first ATG, respectively. PSAPS3ATG refers to the shorter isoform starting at the third ATG. These alignments were carried out using the program Vector NTI (Thermo Fisher Scientific, Waltham, MA, USA). Important data are shown in bold. Some of these scores do not match exactly the results obtained upon blast searches due to differences in settings of the alignment programs.

	Similarity (%)	Identity (%)
Human PSAPL vs. human MTCH2	48.7	35.9
Human PSAPS vs. human MTCH2	**50.7**	**37.3**
Human PSAPS 3ATG vs. human MTCH2	60.9	44.8
Human PSAPL vs. Mtch	38.8	26.2
Human PSAPS vs. Mtch	**40.2**	**27.3**
Human PSAPS 3ATG vs. Mtch	48.1	32.7
Human PSAPL vs. Dm CG10920	34.5	22
Human PSAPS vs. Dm CG10920	**36**	**22.9**
Human PSAPS 3ATG vs. Dm CG10920	29.9	19
Human Mtch2 vs. Mtch	**47.1**	**33.7**
Human Mtch2 vs. Dm CG10920	**33**	**23.5**
Mtch vs. Dm CG10920	**39.9**	**27.1**

**Table 3:** Mtch paralogs in drosophilids.

NCBI RefSeq	Species	Gene ID	Alias ID	Length	Chr	Muller	CDS introns	Current assembly	Location
NP_523869.1	*D. melanogaster*	Mtch	CG6851	316	3L	D	3	Release 6 plus ISO1 MT (GCF_000001215.4)	NT_037436.4 (184626.0.186310)
XP_002034748.1	*D. sechellia*	LOC6610139	GM14313	316	3L	D	3	ASM438219v1 (GCF_004382195.1)	NC_045951.1 (260119.0.261860)
XP_044778913.1	*D. simulans*	LOC6736203	GD13550	316	3L	D	3	Prin_Dsim_3.1 (GCF_016746395.2)	NC_052522.2 (192277.0.194114)
XP_026833781.1	*D. erecta*	LOC6544643	GG14697	316	3L	D	3	DereRS2 (GCF_003286155.1)	NW_020825198.1 (166296.0.168069)
XP_039229778.1	*D. yakuba*	LOC6532190	GE21060	316	3L	D	3	Prin_Dyak_Tai18E2_2.1 (GCF_016746365.2)	NC_052529.2 (205091.0.206612)
XP_001955902.1	*D. ananassae*	LOC6507488	GF24859	318	2R	D	3	ASM1763931v2 (GCF_017639315.1)	NC_057928.1 (26545889.0.26547582)
XP_002065974.1	*D. willistoni*	LOC6643198	GK21061	317	XR	D	3	UCI_dwil_1.1 (GCF_018902025.1)	NW_025814058.1 (2390460.0.2392413)
XP_046869617.1	*D. willistoni*	LOC6651365	GK13597	318	3R	E/F	0	UCI_dwil_1.1 (GCF_018902025.1)	NC_061086.1 (1636555.0.1637828, complement)
XP_026849764.1	*D. persimilis*	LOC6601056	GL16153	314	XR	D	3	DperRS2 (GCF_003286085.1)	NW_020825387.1 (180783.0.184286)
XP_001352469.2	*D. pseudoobscura*	LOC4812188	GA19905	314	XR	D	3	UCI_Dpse_MV25 (GCF_009870125.1)	NC_046683.1 (57227153.0.57228900, complement)
XP_001983612.1	*D. grimshawi*	LOC6556851	GH15483	316	5	D	3	ASM1815329v1 (GCF_018153295.1)	NW_025063240.1 (19935873.0.19937572)
XP_001993136.1	*D. grimshawi*	LOC6566467	GH13246	315	3	B	0	ASM1815329v1 (GCF_018153295.1)	NW_025062900.1 (21574960.0.21576303, complement)
XP_002012054.1	*D. mojavensis*	LOC6586440	GI16759	316	4	D	3	ASM1815372v1 (GCF_018153725.1)	NW_025318899.1 (1095049.0.1096716)
XP_002046438.1	*D. virilis*	LOC6622585	GJ12504	316	3	D	3	DvirRS2 (GCF_003285735.1)	NW_022587374.1 (44903.0.46440)
NP_572408.2	*D. melanogaster*	CG10920	CG10920	397	X	A	0	Release 6 plus ISO1 MT (GCF_000001215.4)	NC_004354.4 (7852481.0.7854244, complement)
XP_002044447.1	*D. sechellia*	LOC6620243	GM11973	397	X	A	0	ASM438219v1 (GCF_004382195.1)	NC_045954.1 (7561254.0.7562924, complement)
XP_002106401.2	*D. simulans*	LOC6725384	GD16147	437^a^/397	X	A	0	Prin_Dsim_3.1 (GCF_016746395.2)	NC_052525.2 (7414923.0.7416670, complement)
XP_001978568.1	*D. erecta*	LOC6551654	GG17610	395	X	A	0	DereRS2 (GCF_003286155.1)	NW_020825209.1 (14217137.0.14218714, complement)
XP_002101236.1	*D. yakuba*	LOC6525401	GE17510	390	X	A	0	Prin_Dyak_Tai18E2_2.1 (GCF_016746365.2)	NC_052526.2 (14038902.0.14040658)
XP_001963867.1	*D. ananassae*	LOC6503933	GF21249	452	XL	A	0	ASM1763931v2 (GCF_017639315.1)	NC_057931.1 (1661245.0.1663572)
XP_002071692.3	*D. willistoni*	LOC6649061	GK18865	399	XL	A	1	UCI_dwil_1.1 (GCF_018902025.1)	NW_025814052.1 (7172991.0.7174536)
XP_026848942.1	*D. persimilis*	LOC6597637	GL14592	383	XL	A	0	DperRS2 (GCF_003286085.1)	NW_020825356.1 (270098.0.271320, complement)
XP_002134490.2	*D. pseudoobscura*	LOC6901822	GA24120	389	XL	A	0	UCI_Dpse_MV25 (GCF_009870125.1)	NC_046683.1 (6139304.0.6147272)
XP_001991312.1	*D. grimshawi*	LOC6565377	GH12126	381	X	A	0	ASM1815329v1 (GCF_018153295.1)	NW_025063692.1 (20979396.0.20980681)
XP_002011063.1	*D. mojavensis*	LOC6585432	GI16212	350	X	A	0	ASM1815372v1 (GCF_018153725.1)	NW_025318667.1 (3374257.0.3375535)
XP_002058107.1	*D. virilis*	LOC6634654	GJ15674	366	X	A	0	DvirRS2 (GCF_003285735.1)	NW_022587403.1 (2115959.0.2117252)

^a^ Probably translated in downstream initiation codon/annotation error.

**Fig. 2:**
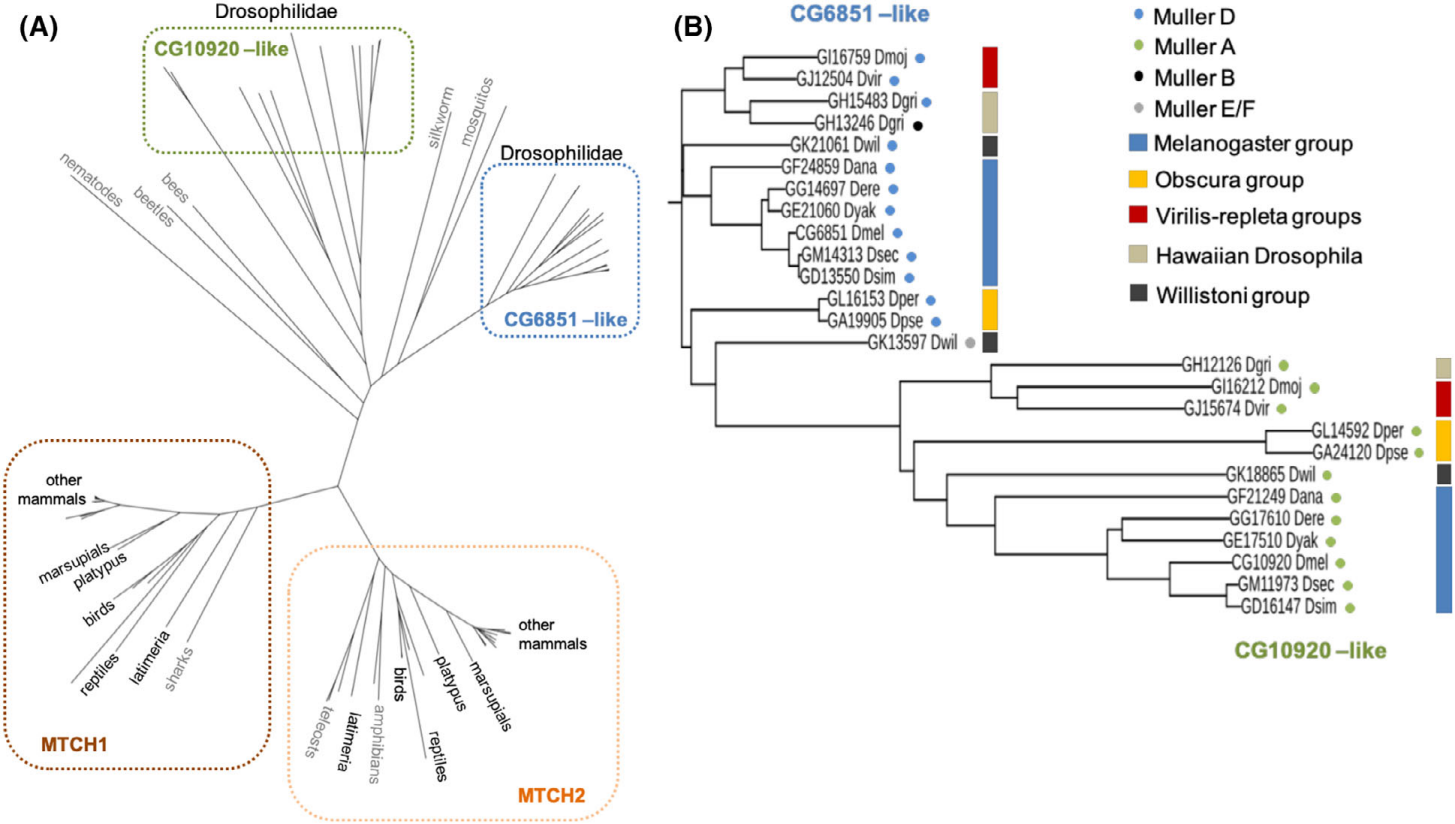
Phylogenetic analysis of Mtch orthologs. (A) Radial phylogenetic tree of Mtch orthologs in vertebrates and invertebrates (including 12 sequenced drosophilids). Most vertebrate species contain two copies of Mtch homologs, one of which related to human MTCH1 and the other to human MTCH2. Exceptions (gray font) are cartilaginous fishes (such as sharks) that contain one MTCH1‐like gene, and bony fishes (teleosts) and amphibians that contain one MTCH2‐like gene. The invertebrate species generally contain one Mtch homolog. However, in drosophilids there are 2‐3 Mtch homologs which we call CG6851‐like and CG10920‐like groups. (B) Detailed phylogram and the syntenic relationships (Muller elements, colored dots) among the Mtch orthologs of the 12 sequenced fly genomes. Because karyotypes vary in different *Drosophila* species, a six‐element Muller element designation (A–F) is used as a standardized notation for the syntenic relationship, i.e. conserved chromosome regions among species. Additionally, a view of the phylogenetic relationships of the species in different groups is shown (colored bars). All drosophilid species shown contain two Mtch orthologs, except Dgri and Dwil which each have three orthologs. The Mtch orthologs can be divided clearly in two phylogenetic/syntenic groups, one being “CG6851‐like” (Muller D) and another “CG10920‐like” (Muller A). The third Mtch orthologs in Dgri (GH13246) and Dwil (Gk13597) are phylogenetically CG10920‐like but located in different Muller elements suggesting more recent gene duplication in these species.

Most (but not all) vertebrate species contain two copies of Mtch homologs, one of which is generally related to human MTCH1 and the other one, to MTCH2 (Fig. 2). To investigate if the fly Mtch orthologs can be grouped similarly, we aligned sequences from Mtch orthologs in 12 sequenced drosophilids with a selected group of annotated vertebrate and invertebrate species. As shown in Fig. 2A, the organization in MTCH1 or MTCH2–like genes is very clear among vertebrates. However, the invertebrate species (which in general contain one MTCH homolog) cannot be distinctively annotated as homologs of MTCH1 or MTCH2. This is also the case with Mtch‐like proteins in drosophilids, where the 2‐3 homologs are clearly separate from each other within invertebrates (CG6851‐like and CG10920‐like groups) but show no specific homology for either human MTCH1 or MTCH2. Further analysis of drosophilid CG6851‐like and CG10920‐like groups confirmed that they form clear phylogenetic/syntenic groups (Fig. 2B) that recapitulate the general phylogeny of drosophilids.

To gain insight into the potential biological significance of the two *Mtch* homologs in flies we investigated where and at which level these genes are expressed using FlyAtlas2 [37]. As shown in Fig. S1, CG6851 was expressed at high levels in all larval (panel A) and adult (panels B and C) tissues examined, whereas CG10920 was basically testis‐specific and not expressed in females or in larval tissues. Although this does not necessarily imply that CG10920 function is dispensable in some specific tissues, we reasoned that CG6851 is a more likely candidate for whole‐organism investigation of Mtch function, and therefore we focused our work on this protein.

### Deletion of *Drosophila* Mtch is lethal in larval or pupal stages

**Fig. 3:**
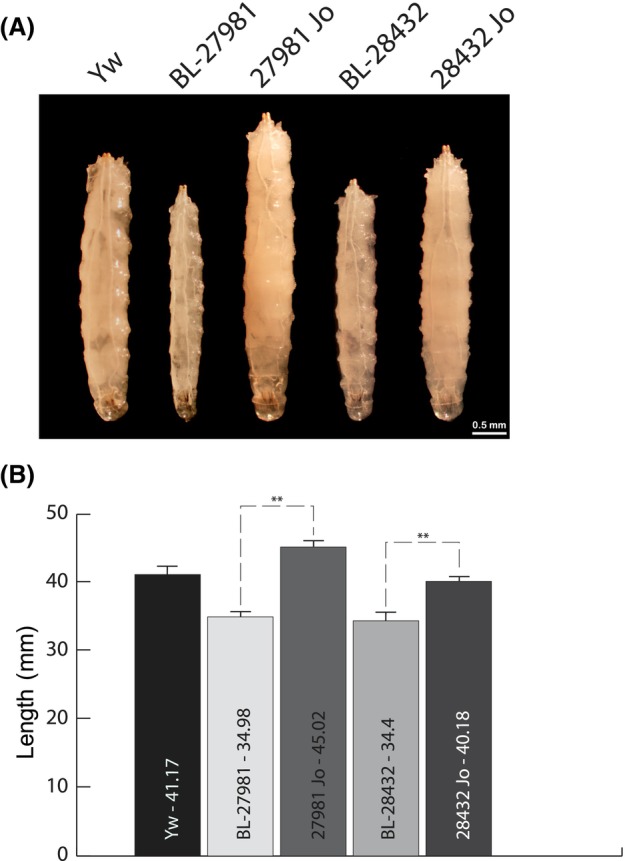
Phenotypes of wild type, mutant and revertant larva. (A) Representative images of each phenotype. (B) Graph indicating the lengths, in mm, of larva (L3) from each type averaged in each case from 6 to 12 individuals (average length is indicated inside each bar). Error bars represent standard deviation. Yw, wild type; BL‐27981 (mutant 1), homozygote larva of this mutant; 27981 Jo, revertant of this mutant; BL‐28432 (mutant 2), homozygote larva of this mutant; 28432 Jo, revertant of this mutant. Statistical analysis was carried out using a two‐tailed test, with *N* = 3, where each *N* represents the average of 6 to 12 larva analyzed on different days. **P < 0.0005. Scale bar in A is 0.5 mm.

To study the role of Mtch in *Drosophila*, we analyzed two different P‐element insertional mutants, lines G8642 and G20854. From here on we will refer to these mutants as mutants 1 and 2, respectively. The precise genotypes of these stocks are shown in Table 1. In mutant 1 P‐element insertion disrupts the gene in the coding sequence, just after leucine at position 57, and is likely to result in complete loss of *Mtch* function. On the other hand, mutant 2 presents the P‐element inserted in the region corresponding to the 5′ UTR of the transcript, which is often associated with a loss or a severe reduction in gene expression. Accordingly, the observed phenotypes are much stronger in mutant 1, further confirming its loss of function (Fig. 3).

Homozygous mutant larvae from both lines showed a clear phenotypic difference with respect to wild type flies (Fig. 3). They were shorter and slimmer, a phenotype often associated with mitochondrial alterations [38, 39]. Homozygous mutant 1 progeny did not survive beyond larval stage 3, whereas mutant 2 homozygous animals reached pupal stages, with very few of them eclosing. In the latter case, the few adult flies that emerged died soon afterward. This is consistent with the expected severity of the mutations based on P‐element insertion sites in the coding region (mutant 1) and 5′ untranslated region (mutant 2).

To confirm that this phenotypic alteration was caused by defects in *Mtch* expression, the P‐elements were genetically removed. Revertants had a wild type phenotype, development and apparent life span, indicating that the described phenotype was caused by *Mtch* inactivation or reduced expression (Fig. 3).

### Knocking out Mtch increases cell apoptosis in wing imaginal discs

**Fig. 4:**
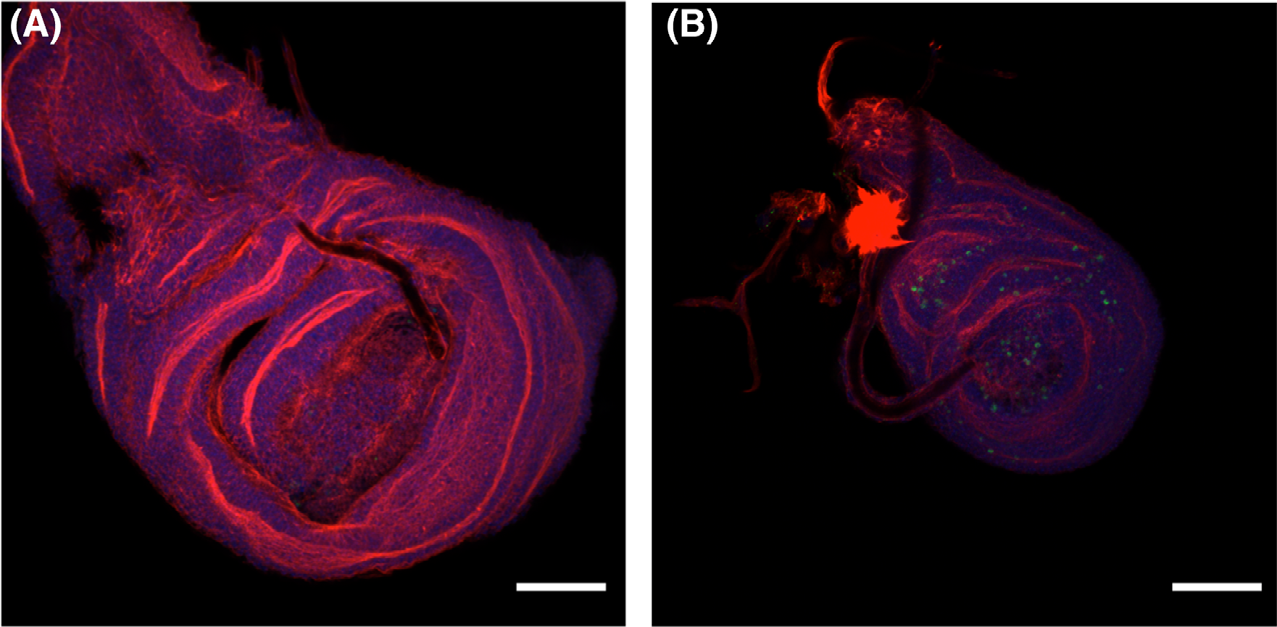
Increase in apoptosis in imaginal discs. Wing imaginal discs were isolated from wild type pupa (A) and from BL‐28432 homozygotes (B), stained for Actin (red) and activated caspase 3 (green) and photographed with a Nikon 90i microscope at 400× magnification. Representative images of at least 12 analyzed imaginal discs are shown. The scale bar is 100 μm.

Human MTCH1 has been reported to induce apoptosis when overexpressed in cultured cells [40] and knocking out *MTCH2* in mice reduces apoptosis mediated by BAX/BAK and induced by tBID [5], therefore we hypothesized that failure to complete development in *Drosophila* upon *Mtch* inactivation could be due to reduced apoptosis during pupation. Since larvae from mutant 2 were able to enter the pupal stage we analyzed wing imaginal discs to identify possible alterations. The discs were stained with phalloidin (red), ToPro3 (blue), and an antibody against activated caspase 3 (green). All imaginal discs analyzed (at least 12 in each experiment) from mutant flies were around half the size of their wild counterparts. Furthermore, staining for activated caspase 3, an indicator of cell apoptosis, showed an increase in apoptotic cell death in mutant flies as compared to wild type counterparts (Fig. 4). These results show that blocking *Mtch* in flies increases apoptosis in imaginal discs, indicating the opposite effect to what has been described in mammalian cells [40].

### Knocking down Mtch1 in schneider cells increases apoptosis

**Fig. 5:**
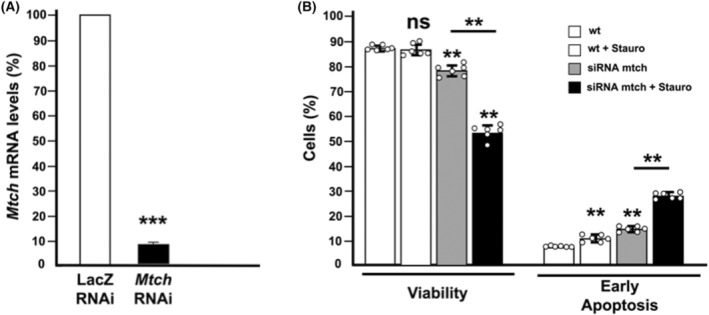
Effect of RNA interference in cultured cells. (A) Mtch mRNA levels under standard and interference situations. The amount of the mRNA was determined by qPCR in exponentially growing Schneider SL2 cells in presence of dsRNA internal to LacZ, as a negative control, or Mtch dsRNA as indicated in Materials and methods. *n* = 6 independent experiments, mean ± SD are shown. ***P value < 0.001. (B) Quantification of viable cells and cells in early apoptosis in a Mtch interference situation and control either in presence and absence of staurosporine. Data are shown as a mean ± SD for *n* = 6 independent experiments. A statistical two‐tailed *t*‐student test was carried out. **P value < 0.01. ns, not significant.

Since many experiments with human MTCH1 have been carried out in cultured cells, we decided to analyze the effects of knocking down *Mtch* directly in *Drosophila* cultured cells, performing RNA interference (RNAi) assays in Schneider cells. Double‐stranded RNA (dsRNA) was designed with a sequence comprising over 200 bp of the 5′ side of *Mtch* mRNA. A dsRNA designed against bacterial *LacZ* was used as negative control [41]. Cells were treated with the dsRNA for 24 h and then divided in three sets. One of these was used for RNA extraction and subsequent analysis by real‐time PCR to measure *Mtch* mRNA levels. The other two sets were used to detect apoptosis in the absence or presence of staurosporine, using annexin‐V and propidium iodide. Upon RNA interference, the levels of *Mtch* mRNA were reduced around 90% with respect to a control (a similar treatment using dsRNA against bacterial LacZ; Fig. 5A). The levels of the mRNAs of three other genes, *Alpha‐tub84B*, *Actin42A*, and *Polr2B*, were also measured and used to normalize *Mtch* mRNA levels among different assays.

Results, shown in Fig. 5B, indicate an increase in apoptotic cells (annexin V‐positive, propidium iodide‐negative) when Mtch levels are reduced with respect to wild type cells. This effect is stronger when cells are treated with the apoptosis inducer staurosporine. This is in perfect agreement with what we observed in imaginal discs by detection of activated caspase 3, indicating that depletion of Mtch increases apoptosis both in cultured cells and in developing flies, which explains the failure to complete pupation.

## Discussion

The function of mammalian MTCH1 has remained elusive for years and only recently have some reports shed light onto its cellular function. We have shown here that significatively decreasing *Drosophila Mtch* function in whole flies or in cultured cells increases apoptosis. The increase in apoptosis appears to be associated with failure of fly metamorphosis, leading to death at larval or pupal stages. These results are striking and unexpected considering the data available from human cultured cells, where overexpression of MTCH1 induces apoptosis.

Of the two fly *Mtch* orthologs, CG6851 and CG10920, the former is the best candidate to be considered the *Drosophila Mtch* since the expression of the latter is confined almost completely to testis and lacks introns, being therefore likely originated by reverse transcription later during evolution. Furthermore, neither *Drosophila* protein could be ascribed to Mtch1 or Mtch2 only based on sequence homology. In fact, the *Drosophila* proteins are not significantly more similar to each other than they are to their mammalian orthologs, although CG6851 was considered the *Drosophila* Mtch2 in a former publication [36], and this is reinforced by DRSC Integrative Orthology Prediction Tool data available in FlyBase (https://www.flyrnai.org/cgi‐bin/DRSC_orthologs.pl). Both human proteins are not very similar themselves (not more than 50% unless the amino terminal region of MTCH1 is truncated).

As a target for tBID in mitochondria, knocking out *MTCH2* is expected to reduce apoptosis initiated by the extrinsic pathway and mediated by tBID, and so was found when it was knocked out conditionally in mouse liver, although the conventional knockouts were embryonically lethal in homozygosis [5]. Lack of MTCH2 did not affect apoptosis induced by other pro‐apoptotic BCL‐2 family members. Zhang *et al*. [28] reported that MTCH1 is involved in apoptosis mediated by the death receptor DR6, which interacts with MTCH1 in mitochondria and with BAX in the cytosol, suggesting that BAX could be transferred from the former to the latter but with no apparent mediation of BID. The question arises whether Mtch could be playing in *Drosophila* a similar role to human MTCH2, as a target for a BID‐like protein. Nevertheless, only two BCL‐2 family members have been reported in *Drosophila*: Debcl (also known as Drob‐1, dBorg‐1 and dBok) and Buffy (also known as dBorg‐2) which is anti‐apoptotic [42, 43, 44, 45, 46, 47]. In one report Debcl was found to be protective against polyglutamine‐induced toxicity in *Drosophila*, and antagonized by Buffy [48]. Both are structurally related to mammalian BOK, containing BH1‐BH3 domains, and Debcl is considered to be the ortholog of BAX/BAK [49] whereas Buffy has a function similar to that of BCL‐XL. No BID‐like protein is present in *Drosophila*, and therefore no candidate for binding to *Drosophila* Mtch has so far been identified (to our knowledge this is the first study of *Drosophila* Mtch). Furthermore, *Drosophila* BCL‐2‐like proteins have been reported to be dispensable for normal development [50]. A later report indicated that Debcl, although not required for gross development and lifespan, was required for pruning cells in the developing central nervous system [49]. Considering these reports and the fact that knocking down *Mtch* in flies is developmentally lethal, it is unlikely that Mtch is acting in *Drosophila* together with any of the two BCL‐2 family members, i.e., its mechanism of action appears to be different from that of MTCH2 in mammals with respect to tBID binding, and also to that of MTCH1 considering its reported interaction with BAX [28]. We must also recall that mammalian MTCH2 was first reported as MIMP, Met‐induced mitochondrial protein, and described to be upregulated by Met‐HGF/SF signal transduction, leading to mitochondrial depolarization [7]. These authors later published that MIMP overexpression reduces Met‐HGF/SF‐induced proliferation and scattering by attenuating and altering the downstream signaling of Met, linking a tyrosine kinase growth factor receptor and a mitochondrial carrier homolog that regulates cellular growth, motility, and tumorigenicity. It is unknown whether *Drosophila* Mtch could be playing a similar role.

MTCH2 has been also involved in lipid metabolism [8, 9], and therefore it is possible that *Drosophila* Mtch could also be involved in some lipid metabolism‐related processes. To our knowledge, no link between the apoptotic function of mammalian MTCH2 and its implication in lipid metabolism has been described, although a possible role for MTCH2 in death related to metabolism regulation cannot be ruled out [51], neither for *Drosophila* Mtch. In support of this possibility, a direct link between glucose metabolism and apoptosis has been reported [52].

The interaction between MTCH2 and tBID at the molecular level has been studied [53]. The MTCH2 peptide that shows the strongest interaction with tBID corresponds to amino acids 140 to 161 in human MTCH2, and 13 out of these 22 amino acids are conserved in *Drosophila* Mtch (see Fig. 1 and Table 2), implying a high degree of conservation (59%) in this stretch, well above the 33.7% identity these two proteins show overall. This level of conservation suggests an important role for this region in protein activity, and it could be involved in interactions with other yet‐to‐know *Drosophila* proteins as it does with tBID in its mammalian counterpart. Nevertheless, we must also consider that human MTCH1 contains 14 conserved amino acids in this stretch of 22, and so far no interaction of tBID with MTCH1 has been reported.

As already mentioned, Zeng *et al*. [27] showed that MTCH1 mediates presenilin 1 (PS1)‐induced apoptosis, with an MTCH‐BAX complex formed upon stimulation by wild type PS1 or gamma‐secretase‐inactive PS1, suggesting that MTCH1 functions as a receptor for BAX under certain apoptotic conditions. In the putative scenario that *Drosophila* Mtch worked as a receptor or anchor for the proapoptotic protein Debcl, we would expect to see reduced, instead of increased, levels of apoptosis upon knocking down Mtch1.

After many years with several researchers trying to identify a definitive function for the MTCH family of proteins it appears that Guna *et al*. [30] have finally pointed to their major function as integrases. Nevertheless, upon consideration of the several reports about these proteins it appears that they may be playing other roles in cells apart from insertases. This was also suggested by Guna *et al*. when they stated that their finding “now provides a molecular explanation for its pleiotropic phenotypes, many of which can be directly ascribed to defects in biogenesis of MTCH2 substrates”, i.e. not all the phenotypes described so far can be directly ascribed to the role of these proteins as insertases. The fact that overexpression of MTCH1, but not MTCH2 (except in one report) is able to induce apoptosis in a BAX/BAK independent manner points in this direction. Also, MTCH1 is able to interact with BAX, but BAX, as well as BCL‐XL, is able to insert into liposomes in the absence of MTCH proteins but depending on tBID [54]. If MTCH proteins are not required for BAX insertion into membranes but BAX interacts with MTCH1 [28] then it appears likely that MTCH1 has a role in apoptosis that does not depend, at least directly, on its insertase activity, which is also supported by the fact that MTCH2 is a target for tBID in mitochondria [5], although tBID can insert into liposomes in the absence of other proteins [54]. MTCH1 fragments are able to induce apoptosis when directed to the outer membrane by fusion to the TMD of BCL‐XL [25], also suggesting other roles for these proteins, as well as the report of Zhang *et al*. [28] that MTCH1 functions as a receptor for DR6 in mitochondria. In the case of MTCH2, although its induction of apoptosis upon overexpression was reported once [55], Guna *et al*. [30] showed that MTCH2 overexpression in human K562 leukemia cells sensitizes them to imatinib‐induced apoptosis, which is dependent on its insertase activity.

In summary, we show here that depletion of *Drosophila* Mtch increases apoptosis in flies as well as in cultured cells, being responsible for developmental failure. These results are in contrast with what has been described for the human orthologs, since overexpression of human MTCH1 in cells induces apoptosis and its depletion has an additive effect to that of MTCH2 on biogenesis of many mitochondrial tail‐anchored proteins. These, and other, findings point towards additional functions of these proteins apart from that as insertases, which prompts further research in this interesting family of proteins.

## Conflict of interest

The authors declare no conflict of interest.

### Peer review

The peer review history for this article is available at https://www.webofscience.com/api/gateway/wos/peer‐review/10.1002/2211‐5463.13742.

## Author contributions

MAF‐M and JAC conceived and supervised the study; JJA supervised and carried out experiments with flies; JMT realized sequence analyses in drosophilids and other organisms; CG, LM‐S and PC carried out most of the experiments; JAC wrote the manuscript with inputs from JJA, JMT and MAF‐M.

## Supporting information


**Fig. S1.** Expression of the fly Mtch homologs in adult flies and larvae.

**Table S1.** Sequence IDs of Mtch orthologs used in this work.

## Data Availability

Data will be made available from the corresponding author upon reasonable request.
