# Supplementary material for: Dysfunction of Drosophila mitochondrial carrier homolog (Mtch) alters apoptosis and disturbs development
Source: FEBS Open Bio. 2023 Dec 19;14(2):276–89. doi: 10.1002/2211-5463.13742 (PMC10839352; doi:10.1002/2211-5463.13742)
Supplement: Supplementary file 1 — Fig. S1. Expression of the fly Mtch homologs in adult flies and larvae. [file FEB4-14-276-s002.pdf]

Fig. S1

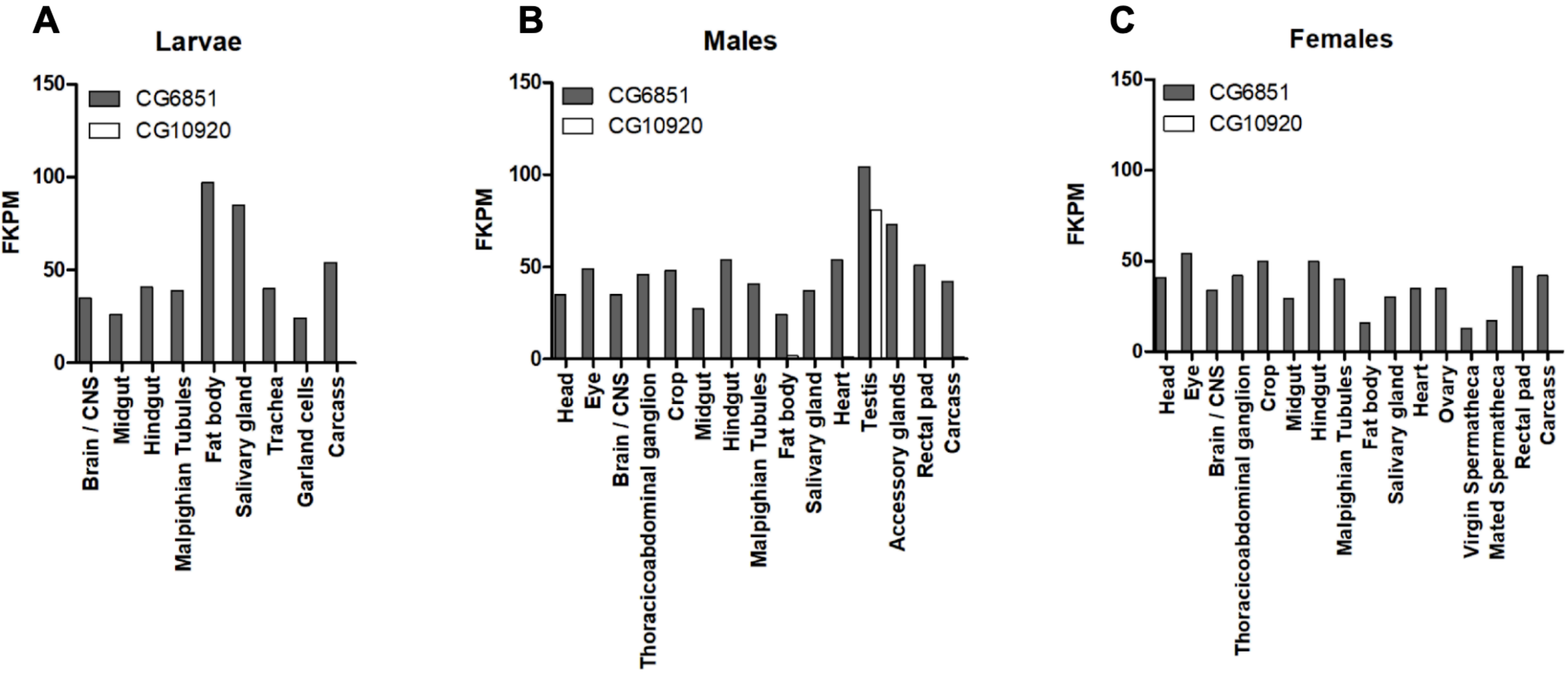

Figure S1. Expression of the fly Mtch homologs in adult flies and larvae. FlyAtlas2 was used to address the level of expression of CG6851 (gray bars) and CG10920 (white bars) in larval (A), adult male (B) and adult female (C) tissues. CG6851 was found to be expressed at high levels in all larval and adult tissues from which the next generation sequencing data was available. CG10920 was found to be testis-specific in adults and expressed at low levels in the fat body. Abbreviations: FKPM, fragments per kilobase of transcript per million mapped reads.
