# Supplementary material for: Dysfunction of Drosophila mitochondrial carrier homolog (Mtch) alters apoptosis and disturbs development
Source: FEBS Open Bio. 2023 Dec 19;14(2):276–89. doi: 10.1002/2211-5463.13742 (PMC10839352; doi:10.1002/2211-5463.13742)
Supplement: Supplementary file 2 — Table S1. Sequence IDs of Mtch orthologs used in this work. [file FEB4-14-276-s001.pdf]

Table S1

Group	Species	MTCH1	MTCH2	Ungrouped
**Amphibians**	*Xenopus tropicalis*		NP_989114.1	
	Aquarana catesbeiana		ACO51744.1	
**Teleosts**	*Danio rerio*		NP_571457.1	
	Ictalurus punctatus		NP_001187386.1	
	Salmo salar		ACM09479.1	
**Latimeria**	*Latimeria chalumnae*	XP_005995489.1	XP_005987017	
**Sharks**	*Callorhinchus milii*	XP_007909270.1		
**Birds**	*Gallus gallus*	XP_046789145.1	NP_990139.1	
	Meleagris gallopavo	XP_019478700.2	XP_003206195.1	
	Taeniopygia guttata	XP_041577150.1	XP_030131272	
**Reptiles**	*Alligator mississippiensis*	XP_006266255.1	XP_006270887	
	Chelonia mydas	XP_037741318.1	XP_037755195.1	
**Other mammals**	*Homo sapiens*	NP_001258570.1	NP_055157.1	
	Mus musculus	NP_063933.1	NP_062732.1	
	Rattus norvegicus	NP_001094303.1	NP_001099958.2	
	Canis lupus familiaris	XP_038409833.1	XP_005631288.2	
	Macaca mulatta	XP_001116955.1	NP_001307433.1	
	Pan troglodytes	XP_009449441.1	XP_054517341.1	
	Bos taurus	NP_001096775.1	NP_001304265.2	
	Sus scrofa	XP_020953648.1	XP_003353925.2	
**Marsupials**	*Monodelphis domestica*	XP_001379087.2	XP_007497502.1	
**Platypus**	*Ornithorhynchus anatinus*	XP_001520336.1	XP_003430581	
**Mosquitos**	*Anopheles gambiae*			XP_308794.4
	Aedes aegypti			XP_001650362.1
**Silkworm**	*Bombyx mori*			NP_001036860.1
**Bees**	*Apis mellifera*			XP_006569591.1
**Beetles**	*Tribolium castaneum*			XP_967504.2
**Nematodes**	*Caenorhabditis elegans*			NP_495545.3
**Drosophilidae**	*Drosophila (D. melanogaster)*			NP_523869.1; NP_572408.2
	Drosophila (D. simulans)			XP_002083098.1; XP_002106401.1
	Drosophila (D. sechellia)			XP_002034748.1; XP_002044447.1
	Drosophila (D. yakuba)			XP_002092964.1; XP_002101236.1
	Drosophila (D. erecta)			XP_001970979.1; XP_001978568.1
	Drosophila (D. ananassae)			XP_001955902.1; XP_001963867.1
	Drosophila (D. willistoni)			XP_002065974.1; XP_002071692.1; XP_002072613.1
	Drosophila (D. virilis)			XP_002046438.1; XP_002058107.1
	Drosophila (D. mojavensis)			XP_002012054.1; XP_002011063.1
	Drosophila (D. pseudoobscura)			XP_001352469.2; XP_002134490.1
	Drosophila (D. grimshawi)			XP_001983612.1; XP_001993136.1; XP_001991312.1
	Drosophila (D. persimilis)			XP_002026111.1; XP_002022740.1

Table S1. Sequence IDs of Mtch orthologs used in this work.
